# Sex-specific associations of muscular fitness with overall academic performance and specific school subjects in adolescents: the EHDLA study

**DOI:** 10.3389/fpsyg.2024.1396163

**Published:** 2024-07-12

**Authors:** David Manzano-Sánchez, Héctor Gutiérrez-Espinoza, José Francisco López-Gil

**Affiliations:** ^1^Department of Didactics of Musical, Plastic and Corporal Expression, Faculty of Education and Psychology, University of Extremadura, Badajoz, Spain; ^2^Análisis Comportamental de la Actividad Física y el Deporte (ACAFYDE), Universidad de Extremadura, Badajoz, Spain; ^3^One Health Research Group, Universidad de Las Américas, Quito, Ecuador; ^4^Department of Communication and Education, Universidad Loyola Andalucía, Seville, Spain

**Keywords:** muscular fitness, physical education, cognitive performance, education, healthy lifestyle

## Abstract

**Introduction:**

Overalll, muscular fitness and academic performance are two variables widely studied in the literature. However, evidence on the relationship between muscular fitness and specific school subjects (e.g., physical education), as well as their differences by sex, is scarce.

**Objective:**

The aim of this study was to examine the associations between muscular fitness and overall academic performance and between muscular fitness and specific subjects (i.e., language, math, foreign language, and physical education) in a sample of secondary Spanish school students.

**Methods:**

For the present study, a sample of 766 students (45% boys, aged 12-17 years). From the Eating Healthy and Daily Life Activities (EHDLA) study was used. The Assessing the Levels of Physical Activity and Fitness (ALPHA-FIT) battery was used to determine handgrip strength and lower body muscular strength. The performance of the different school subjects was provided by the school centers.

**Results:**

Overall, for both boys and girls, higher muscular fitness was associated with greater academic results, with the greatest differences in physical education. However, only significant differences were identified for girls.

**Discussion and conclusion:**

Global muscular fitness is important for contributing to cognitive development, both in physical education and in the rest of the subjects of the educational curriculum for adolescents. At the same time, although the association appears to follow the same trend for both sexes, the results seem more evident for girls.

## 1 Introduction

In adolescents, the relationship between physical activity and academic performance has received considerable attention (Haapala, 2012; Kalantari and Esmaeilzadeh, 2016) in the same way as physical fitness and academic performance (Suchert et al., 2016; Ruiz-Ariza et al., 2017) or muscular fitness (MF) and academic performance (Muntaner-Mas et al., 2018). In this field, the benefits of resistance training on the neuromuscular system are well documented, and there is increasing evidence that it can induce neuroplastic changes in the nervous system and improve cognitive and mental function (Chow et al., 2021). The mental health benefits of aerobic-based physical activity have been reported in the literature (Kandola et al., 2016; Northey et al., 2018). Specifically, strength training is an important variable for the improvement of cognitive performance, including a transcendental aspect that is the deterioration in memory and central executive functioning with age (Frith and Loprinzi, 2018). According to a systematic review with meta-analysis by Robinson et al. (2023), resistance training may have a positive effect on overall cognitive, academic and on-task behaviors in schoolchildren; however, MF is associated with cognition and academic performance. Other studies, such as Gordon et al. (2017) and Bennie et al. (2020), have pointed out how strength training also has benefits on a psychological level (e.g., mental health), reducing symptoms of depression and anxiety and hence another essential aspect to consider.

According to the recommendations of the World Health Organization (2020), it is necessary to include strength training on a regular basis to achieve health benefits. According to these recommendations, children and adolescents between 5 and 17 years old should engage in at least 60 minutes a day of moderate to vigorous physical activity and participate in 3 days a week of resistance work to strengthen their muscles and bones. This training could improve physical fitness, cardiometabolic health, bone health, mental health, and cognitive outcomes such as executive function (Muñoz-Parreño et al., 2021; Chen et al., 2023) or academic performance (Xu and Sun, 2023). In this field, physical fitness includes cardiorespiratory endurance, MF, muscular endurance, and flexibility (Faigenbaum et al., 2020). García-Hermoso et al. (2019) reported that MF had a negative association with adiposity and cardiometabolic disease risk score and a positive association with bone health. In support of this notion, scientific evidence has shown that strength training is beneficial for the development of children and young people, and many benefits are recommended to include it in physical education lessons through pedagogical activities and adapted strategies (Dos Santos et al., 2022), considering that physical fitness has an important role in mediating the association between physical activity and academic achievement via higher levels of fitness, including aerobic performance and strength (Syväoja et al., 2018).

Similarly, it is necessary to specify the definition of academic performance. Academic performance is assessed by computing the grade point average of all school subjects (Suchert et al., 2016; Hsieh et al., 2018; Martinez-Zamora et al., 2021), the grade obtained in language, maths and foreign language (English), and the grade point average of these subjects (Muntaner-Mas et al., 2021; Tapia-Serrano et al., 2021) or standardized tests (Raine et al., 2018). Other study (Manzano-Sánchez et al., 2020) has assessed physical education performance with the grades reported at the end of the course and in each quarter. Moreover, academic performance is related to physical fitness, considering body mass index (BMI), MF, aerobic endurance of primary school students (Xu and Sun, 2023), aerobic endurance, MF, and agility (Hansen, 2017), and junior high school students aged 12 to 15  years (Hsieh et al., 2018). Finally, MF is a variable associated with cognitive performance, but this effect appears to be independent of age, physical activity and other covariates (Frith and Loprinzi, 2018).

Previous studies have examined the relationship between MF and academic performance (Padilla-Moledo et al., 2012; Esteban-Cornejo et al., 2014; García-Hermoso et al., 2017; Gil-Espinosa et al., 2020). For instance, some papers have examined the relationships between both upper and lower body muscular strength and academic performance (Padilla-Moledo et al., 2012; Esteban-Cornejo et al., 2014; Muntaner-Mas et al., 2018) and between lower body muscular strength and other factors (García-Hermoso et al., 2018; Gil-Espinosa et al., 2020) in the young population. Furthermore, only the studies by Gil-Espinosa et al. (2020) and Muntaner-Mas et al. (2018) included academic performance in physical education. Handgrip strength is commonly used as a proxy of total MF because it is strongly correlated in adolescents (Wind et al., 2010; Weedon et al., 2022; De Almeida Santana et al., 2023). Moreover, equal consideration of lower body muscular strength is also relevant since it is strongly associated with other tests that assess both upper and lower body muscular strength (Castro-Piñero et al., 2010). Therefore, combining the handgrip strength test and the standing long jump could provide a more comprehensive measure of global MF than evaluating these components separately. To our knowledge, there are no previous studies evaluating MF (combining both upper and lower body muscular strength) with performance in some specific school subjects (including physical education). Only the study by Muntaner-Mas et al. (2018) assessed the relationship between MF (including both upper and lower muscular strength) and academic performance in physical education. However, these authors did not discuss this specific result and did not stratify the analyses by sex. Therefore, the aim of this study was to examine the association between MF and academic performance according to the grade achieved in language, math, foreign language and physical education and the average global score in a sample of Spanish secondary school students.

## 2 Methods

### 2.1 Design and procedure

In this study, we conducted a secondary analysis of data from the Eating Healthy and Daily Life Activities (EHDLA) project, which was previously described (López-Gil, 2022). The sample consisted of adolescents aged 12–17 years who were enrolled as students in three secondary schools in this region during the 2021–2022 academic year. The EHDLA study used a simple random sampling technique to determine the proportion of adolescents with overweight/obesity from the *Valle de Ricote* (Region of Murcia, Spain). With a 95% confidence interval (CI), an estimated prevalence of overweight and obesity (40.0%), a 3% margin of error, and a 10% nonresponse rate, the minimum sample size required was 1,138. For this study, the sample was restricted to adolescents who provided complete information on the variables of interest. To be eligible for the study, participants had to be enrolled in physical education classes and not have any medical conditions that limited their physical activity or required special attention, be taking any medications, lack parental authorization to participate, or decline to participate in the research project. Participants who met these criteria were excluded from the study.

The Bioethics Committee at the University of Murcia, as well as the Ethics Committee of the Albacete University Hospital Complex and the Albacete Integrated Care Management, granted approval for this study (approval IDs: 2218/2018, 2021-85). Written permission was obtained from the parents or legal guardians of the adolescents who participated in the study. The participants were informed about the study’s objectives and the assessments and questionnaires that would be administered. Additionally, the adolescents provided written consent to participate in the study following a discussion of study-related procedures. The research adhered strictly to the ethical principles outlined in the Helsinki Declaration.

### 2.2 Participants

Of the original 1,378 adolescents involved in the EHDLA study, 118 participants (8.6%) were excluded due to missing data on academic performance. Furthermore, 67 (4.9%) participants were removed from the analysis because of missing information on handgrip strength, and 14 (1.0%) were removed because of a long standing jump. Additionally, further exclusions were made for participants with incomplete data on BMI (*n* = 51; 3.7%), energy intake (*n* = 319; 23.1%), sleep duration (*n* = 36; 2.6%), or physical activity (*n* = 7; 0.5%). Consequently, this secondary cross-sectional study included a sample of 766 adolescents (45.0% boys).

### 2.3 Measures

#### 2.3.1 Muscular fitness

The Assessing the Levels of Physical Activity and Fitness (ALPHA-FIT) battery (Ruiz et al., 2011) was utilized to evaluate MF in young individuals. To specifically assess upper body strength, a hand dynamometer with an adjustable grip (TKK 5401 Grip D, Takei, Tokyo, Japan) was used for the handgrip strength test. Before performing the test, the participants received a brief tutorial and verbal instructions. The dynamometer was adjusted based on the child’s hand size, and the test was conducted while the child stood in a position with their elbow extended and wrist in a neutral position. Participants were encouraged to exert their maximum handgrip strength for at least 2 s. The test was performed twice for each hand, and the highest score from each hand was recorded. The average of the highest scores from both hands was then used for further analysis.

To evaluate lower body muscular strength, the standing long jump test was performed. Participants began behind a designated line with their feet together and jumped forward as far as possible. The distance was measured from the take-offline to the point where the back of the heel closest to the take-offline contacted the floor. The test was performed twice, and the highest score in centimeters was selected for analysis. On the other hand, individual z scores were calculated for both handgrip strength and jumping, and MF was determined as the average of these two z scores.

#### 2.3.2 Academic performance

The assessment of academic performance involved determining the cumulative grade point average (GPA) for all participants. This was done by adding up the numerical grades for each subject and then dividing the total by the number of subjects. The resulting calculation yielded a final average score that ranged from 0 (the lowest possible score) to 10 (the highest possible score). Furthermore, academic performance in certain specific school subjects (language, math, foreign language, and physical education) was also considered.

#### 2.3.3 Covariates

The adolescents reported their sex and age, and their socioeconomic status was assessed using the Family Affluence Scale-III (FAS-III) (Currie et al., 2008). The FAS-III consists of six items related to family possessions and amenities, which are scored on a scale from 0 to 13, with higher scores indicating a higher socioeconomic status. Adolescents were asked about their usual bedtime and wake-up times on both weekdays and weekends to determine their overall sleep duration, which was calculated using the formula [(average sleep duration on weekdays × 5) + (average sleep duration on weekends × 2)] divided by 7. This methodology for estimating overall sleep duration has been applied in several previous studies (Lauderdale et al., 2008; Kim et al., 2022; Echevarria et al., 2023). The Spanish Youth Activity Profile Physical Questionnaire (Segura-Díaz, et al., 2021), which covers a 7-day period and includes 15 items categorized into sections such as out-of-school activities, school-related activities, and sedentary habits, was used to assess physical activity and sedentary behavior.

Energy intake was estimated from a self-administered food frequency questionnaire that had been validated for the Spanish population (Rodríguez et al., 2008), which measured portions consumed in the last month. The body weight and height of the adolescents were measured using an electronic scale with an accuracy of 0.1 kg (Tanita BC-545, Tokyo, Japan) and a portable height rod with an accuracy of 0.1 cm (Leicester Tanita HR 001, Tokyo, Japan), respectively. Subsequently, BMI was computed by dividing the participants’ body weight in kilograms by the square of their height in meters.

### 2.4 Statistical analysis

To assess the normality of the variables, we employed visual techniques such as density and quantile–quantile plots, as well as the Shapiro–Wilk test. For categorical variables, descriptive statistics were calculated for both the count (*n*) and the percentage (%) of occurrences within each category of MF status. For continuous variables, descriptive statistics included the median and the interquartile range (IQR) due to the nonnormal distribution of the variables. While a preliminary analysis revealed a nonsignificant interaction effect between sex and handgrip strength (*p* = 0.105) concerning GPA, a significant interaction effect was identified between sex and standing long jump (*p* = 0.043). Consequently, the main analyses were stratified by sex. We utilized generalized additive models (GAMs) to investigate the associations between MF and academic performance in adolescents without considering the specific nature of the relationship. The restricted maximum likelihood method was applied for selecting smoothness (Wood, 2011), and thin plate regression spline smoothers (Marra and Wood, 2011) were used as a shrinkage approach. The effective degrees of freedom (*edf*) of the GAM were used to quantify the degree of nonlinearity in the relationship. To determine the associations between MF status (i.e., low MF, medium MF, high MF) and academic performance while adjusting for several covariates, generalized linear models (GLMs) were constructed. A nonparametric bias-corrected and accelerated (*BCa*) bootstrap method with 1,000 samples was used for this analysis. Following the analysis, multiple comparisons were corrected for using the false discovery rate *p* value method developed by Benjamini and Hochberg (1995). All models were adjusted for various covariates, such as age, socioeconomic status, overall sleep duration, physical activity, sedentary behavior, energy intake, and BMI. All the statistical analyses were conducted using R statistical software (version 4.3.2) developed by the R Core Team in Vienna, Austria, and RStudio (version 2023.09.1 + 494) from Posit in Boston, MA, United States. The threshold considered for statistical significance was a *p* < 0.05.

## 3 Results

Table 1 shows the descriptive data of the study participants based on their MF status and stratified by sex. For both sexes, the highest GPA was observed for adolescents with a high MF (boys: median = 6.8, IQR = 2.8; girls: median = 7.6, IQR = 2.2). The lowest GPA was identified for adolescents with low MF (median = 6.2, IQR = 2.3). Conversely, in girls, the lowest GPA was found in those with medium MF (median = 6.9, IQR = 2.7).

**Table 1:** Descriptive data of the study participants according to their muscular fitness status by sex.

	Boys (*n* = 345)			Girls (*n* = 421)		
Variable	Low MF	Medium MF	High MF	Low MF	Medium MF	High MF
Age (years)	13.0 (1.8)	13.0 (1.0)	15.0 (2.0)	13.0 (2.0)	14.0 (2.0)	15.0 (3.0)
FAS-III (score)	8.0 (2.0)	8.0 (3.0)	9.0 (2.5)	8.0 (2.0)	8.0 (2.0)	9.0 (3.0)
YAP-S PA (score)	2.7 (0.9)	2.6 (0.9)	2.6 (1.0)	2.6 (0.8)	2.6 (0.7)	2.7 (1.0)
YAP-S SB (score)	2.6 (0.8)	2.6 (0.8)	2.8 (1.0)	2.6 (0.8)	2.4 (0.8)	2.4 (0.8)
Sleep duration (minutes)	514.3 (67.5)	510.0 (64.3)	488.6 (66.4)	512.1 (82.5)	492.9 (69.6)	484.3 (72.9)
Energy intake (kcal)	2473.6 (1467.9)	2400.9 (1351.0)	2734.6 (1826.9)	2516.7 (1359.3)	2583.1 (1523.4)	2752.0 (1437.3)
BMI (kg/m^2^)	22.3 (7.2)	22.0 (6.3)	21.7 (6.4)	21.4 (6.4)	21.3 (4.2)	21.7 (5.2)
Handgrip mean (kg)	19.2 (5.1)	26.0 (7.4)	35.8 (8.2)	17.0 (4.1)	20.7 (3.9)	25.0 (5.8)
Standing long jump (cm)	126.0 (30.1)	157.0 (25.0)	192.0 (30.2)	109.8 (23.2)	133.0 (20.8)	156.5 (28.5)
MF (z score)	−1.8 (0.9)	−0.2 (0.8)	1.9 (1.1)	−1.5 (1.0)	−0.1 (0.7)	1.5 (1.3)
Language (score)	6.0 (3.0)	6.0 (2.5)	6.0 (4.0)	7.0 (4.0)	7.0 (2.0)	7.0 (3.0)
Maths (score)	6.0 (3.0)	6.0 (3.5)	6.0 (4.0)	6.0 (4.0)	6.0 (4.0)	6.0 (3.0)
Physical education (score)	7.0 (2.0)	7.0 (3.0)	8.0 (3.0)	7.0 (2.0)	7.0 (2.0)	8.0 (2.0)
Foreign language (score)^†^	6.0 (3.0)	6.0 (2.0)	6.0 (3.0)	7.0 (3.0)	7.0 (3.0)	7.0 (3.0)
GPA (score)^**‡**^	6.2 (2.3)	6.5 (2.5)	6.8 (2.8)	7.0 (2.4)	6.9 (2.7)	7.6 (2.2)

The data are expressed as medians and interquartile ranges. BMI, body mass index; FAS-III, Family Affluence Scale-III; GPA, grade point average; MF, muscular fitness; PA, physical activity; SB, sedentary behavior; YAP-S, Spanish Youth Active Profile. ^†^English as a foreign language. ^‡^The GPA was calculated as the average of all the measurements taken by the adolescents.

**Figure 1:**
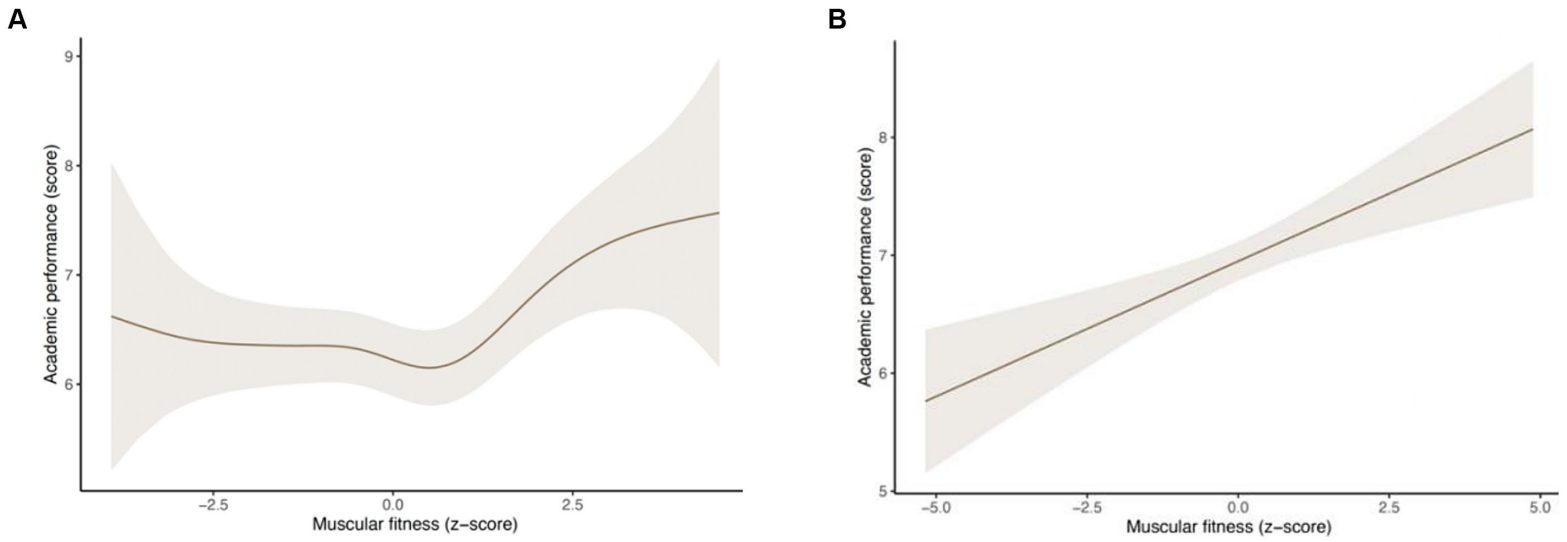
**(A)** Estimated marginal means from generalized additive models for grade point average based on muscular fitness among male adolescents. **(B)** Estimated marginal means from generalized additive models for grade point average based on muscular fitness among female adolescents.

Figure 1 shows the estimated marginal means from the GAMs for GPA based on MF (z score) by sex. The approximate significance of smooth terms was significant for both sexes (*p* < 0.05). A nonlinear association between MF and GPA was identified. Overall, in both sexes, participants with the highest MF z scores had the greatest GPA scores.

Table 2 displays the estimated marginal means of different school subjects (i.e., language, math, English, and physical education) based on MF status by sex. Overall, adolescents with high MF showed the highest means for all the individual school subjects examined. The only exception was for language in boys. For boys, significant differences were found for physical education between adolescents with medium MF (mean (*M*) = 6.7, 95% *BCa* CI 6.3–7.1) and those with high MF (*M* = 7.5, 95% *BCa* CI 7.1–7.9). In girls, significant differences were also identified for physical education between adolescents with low MF (*M* = 6.9, 95% *BCa* CI 6.6–7.2) and their counterparts with high MF (*M* = 7.6, 95% *BCa* CI 7.3–7.9).

**Table 2:** Estimated marginal means of different school subjects according to their muscular fitness status by sex.

	Boys (*n* = 345)			Girls (*n* = 421)		
Subject	Low MF	Medium MF	High MF	Low MF	Medium MF	High MF
Language (score)	6.2 (5.8–6.7)	5.9 (5.5–6.3)	6.1 (5.6–6.6)	6.6 (6.2–7.0)	6.7 (6.4–7.1)	7.2 (6.8–7.6)
Maths (score)	5.5 (5.0–6.0)	5.4 (4.9–5.9)	6.1 (5.6–6.6)	5.7 (5.3–6.1)	5.9 (5.5–6.3)	6.3 (5.9–6.7)
Foreign language (score)^†^	6.0 (5.6–6.4)	5.9 (5.5–6.3)	6.1 (5.7 to 6.6)	6.3 (6.0 to 6.7)	6.5 (6.1 to 6.8)	6.7 (6.4–7.1)
Physical education (score)	6.9 (6.5–7.3)	6.7 (6.3–7.1)	7.5 (7.1–7.9)^b^	6.9 (6.6–7.2)	7.1 (6.8–7.4)	7.6 (7.3–7.9)^a^

**Figure 2:**
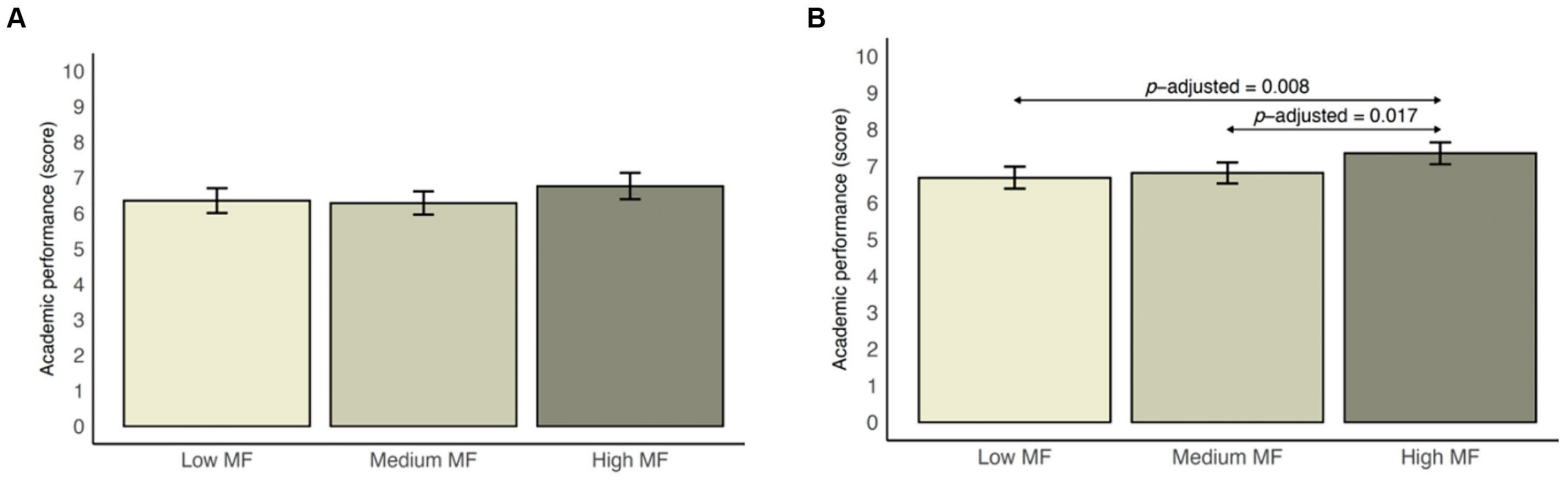
**(A)** Estimated marginal means from generalized linear models with bias-corrected and accelerated bootstrap 95% confidence intervals according to muscular fitness status for male adolescents. **(B)** Estimated marginal means from generalized linear models with bias-corrected and accelerated bootstrap 95% confidence intervals according to muscular fitness status for female adolescents. The data are expressed as estimated marginal means (bars) and bias-corrected and accelerated bootstrapped 95% confidence intervals (lines). Adjusted for sex, age, socioeconomic status, adherence to the Mediterranean diet, energy intake, physical activity, sedentary behavior, overall sleep duration, and body mass index. MF, muscular fitness. †English as a foreign language. aStatistically significant difference with low muscular fitness (p < 0.05); bStatistically significant difference with medium muscular fitness (*p* < 0.05).

The estimated marginal means of GPA and their *BCa* bootstrapped 95% CIs according to MF status (stratified by sex) are shown in Figure 2. For boys, although no significant differences were found among the different MF statuses, the highest GPA mean was identified in adolescents with high MF (*M* = 6.8, 95% *BCa* bootstrapped CI 6.4–7.1). For girls, the highest mean GPA was observed for those with high MF (*M* = 7.4, 95% *BCa* bootstrapped CI 7.1–7.7), with significant differences between these participants and their counterparts with low MF (*p* = 0.008) and those with medium MF (*p* = 0.017).

## 4 Discussion

The aim of this study was to examine the associations between MF (upper and low strength) and overall academic performance and individual school subjects (i.e., language, math, foreign language, and physical education). To our knowledge, only one study from Muntaner-Mas et al. (2018) assessed the relationship between MF (including both upper and lower body muscle strength simultaneously) and academic performance in physical education. Although the relationship between MF and academic performance was not linear, we observed that boys and girls with the highest levels of MF obtained higher school grades. The almost linear relationship we observed in girls seems to suggest that the potential benefits of having a higher MF in terms of overall academic performance could be greater in girls. Dividing MF according to tertiles, we observed that there were significant differences in overall academic performance, but only for girls.

In terms of specific subjects, we observed that the largest differences occurred in physical education grades. These results are in line with the scientific literature, which suggests that a higher MF is related to greater academic performance (de Almeida Santana et al., 2023; Robinson et al., 2023). Conversely, another review concluded that this relationship was not evident (Segura-Díaz et al., 2021). The relationship between greater MF and greater academic performance in adolescents may be linked to various interconnected factors. MF is correlated with several variables, such as sport participation, active transport and physical activity. However, this variable has been studied to the greatest extent and has been shown to be the most influential variable in MF, especially during vigorous exercise (Smith et al., 2019). For this reason, although the exact connection may vary from individual to individual, there are several possible reasons for these findings related to physical exercise.

Physical exercise (including strength training) has been shown to have significant benefits on both academic performance (Cappelen et al., 2017; Li et al., 2021) and cognitive function (Biddle et al., 2019; Alexander and Allendorfer, 2023; Logan et al., 2023). It is possible that physical exercise increases neurogenesis and angiogenesis, increases mitochondrial biogenesis and antioxidant enzyme activity, and provides energy to the brain because of the production of lactate; additionally, physical exercise allows the release of anti-inflammatory cytokines that reduce neuroinflammation (Chen and Nakagawa, 2023). Another possible explanation is that this relationship could be related to insulin-like growth factor-1 (IGF-1). Resistance training increases the release of IGF-1, which can promote the growth and survival of neurons and improve brain plasticity and executive functions (Cassilhas et al., 2007; Xu and Sun, 2023).

On the other hand, physical exercise seems to reduce stress, depression and anxiety in the general population (McDowell et al., 2019; Singh et al., 2023), in children and young people (Carter et al., 2021), and in people with mental health disorders or chronic diseases (Singh et al., 2023). Supporting this notion, Teferi (2020) and Ferreira et al. (2021) concluded that physical exercise could influence anxiety or depression with a moderate-small effect and could influence academic performance. However, caution is required to interpret this hypothesis because the effect sizes ranged from small to moderate, and considerable inconsistency in results among studies was identified (Ferreira et al., 2021).

The benefits of strength training for children and adolescents include improvements in health, fitness, injury prevention and rehabilitation (Stricker et al., 2020) and physical literacy (Zwolski et al., 2017), which may have important effects on the academic performance of students (Domínguez-Martín et al., 2024). The relationship between physical activity programs and physical activity level could increase social–emotional skills (Taylor et al., 2017) with opportunities to interact and communicate between students (Goh et al., 2022), and these social–emotional skills may be transferable to the academic environment, improving adolescents’ ability to manage time, set goals, and collaborate with others. Therefore, establishing healthy routines such as regular strength training could include healthy habits such as regular sleep habits (Urrila et al., 2017; Santos et al., 2023) or paying attention to nutrition, which plays a crucial role in facilitating the smooth growth and development of brain organs (Athaya et al., 2023) and academic performance.

Regarding sex, Álvarez-Bueno et al. (2020) noted that although physical fitness was positively associated with academic performance in both sexes, these associations were stronger in boys than in girls. Similarly, Janak et al. (2014) also reported that regardless of sex and educational stage, students with greater physical fitness showed greater performance in cognitive aptitude tests, but there were no differences between the sexes. Conversely, Padilla-Moledo et al. (2012) did not identify an interaction effect between sex and MF in relation to academic performance. These contradictory results may be due to the way participants’ physical fitness was assessed. Another possible explanation for the difference in our study due to the more favorable results in girls could be that stress and anxiety levels are generally greater in females than in males (Gao et al., 2020).

In relation to specific subjects, our results agreed with a previous study that observed a positive relationship between MF and physical education (Muntaner-Mas et al., 2018) but disagreed with other (Gil-Espinosa et al., 2020). However, Gil-Espinosa et al. (2020) included physical education with the rest of the participants separately, and the relationship between MF and academic performance was not significant in physical education, but it was significant in other subjects (i.e., math). On the other hand, Muntaner-Mas et al. (2018) showed that cardiorespiratory fitness was associated with overall academic performance, but only MF was individually linked with physical education (and math). One possible explanation could be related to the fact that this subject contributes to increasing MF (García-Hermoso et al., 2020) or because individuals with a higher MF may have greater interest in the subject that revolves around the body and movement according to the Committee on Physical Activity and Physical Education in the School Environment, Food and Nutrition Board, and Institute of Medicine (2013). As previously mentioned, greater physical literacy is related to greater academic performance (Domínguez-Martín et al., 2024), which could also explain this result.

### 4.1 Main limitations and strengths of this study

This was a cross-sectional study, and a cause-and-effect relationship could not be established. Nevertheless, the associations found in cross-sectional studies could serve as a reference for designing more specific and effective interventions aimed at increasing academic performance in adolescents. Similarly, although the analyses were adjusted for the level of physical activity outside schools and during weekends through the YAP-S questionnaire, it is possible that some sports activities were not completely captured with the use of this questionnaire, which could also influence the association between physical fitness and academic performance in adolescents (Donnelly et al., 2016). Furthermore, although academic performance was provided by the school centers, this variable is not free of biases from the teachers and could be assessed with more cognitive-oriented tests, such as intelligence tests. Conversely, a strength of the present study is the way of measuring MF, given that most of the studies did not combine both upper and lower body muscular strength, which could offer less accurate results. Combining upper body muscular strength (e.g., handgrip strength test) and lower body muscular strength (e.g., standing long jump test) is suitable for evaluating MF in children, adolescents, and young adults in clinical and educational research (Wind et al., 2010). Finally, the use of GAMs allows the capture of nonlinear relationships in the data without requiring a predefined mathematical structure.

### 4.2 Future research

Longitudinal studies in which the results of this study can be verified with large population samples are recommended. At the same time, it is interesting to apply intervention protocols during school classes to improve fitness in general and carry out physical activity with methodologies such as active breaks (Muñoz-Parreño et al., 2021) given the relationships that have been shown to have with physical and mental health, as well as academic performance. On the other hand, the results between physical fitness and academic performance differ according to the test characteristics and demographic sample examined, which makes it necessary to expand the assessment of this relationship to other age phases and carry out studies using similar tests to reach more robust conclusions (Álvarez-Bueno et al., 2020).

## 5 Conclusion

The main conclusions of the study corroborate that MF is related to academic performance both in general and specifically in the context of physical education. At the same time, although the association appears to follow the same trend for both sexes, the results seem more evident for girls. However, it is interesting to consider future studies that include other variables, such as BMI or the level of aerobic fitness, given that various investigations have considered these two variables, making it necessary to use valid instruments such as manual dynamometry or a long jump to assess strength performance in students.

## Data availability statement

The raw data supporting the conclusions of this article will be made available by the authors, without undue reservation.

## Ethics statement

Ethics Committee of the Albacete University Hospital Complex and the Albacete Integrated Care Management, granted approval for this study (approval IDs: 2218/2018, 2021-85). The studies were conducted in accordance with the local legislation and institutional requirements. Written informed consent for participation in this study was provided by the participants’ legal guardians/next of kin.

## Author contributions

DM-S: Writing – original draft, Methodology, Conceptualization. HG-E: Writing – review & editing. JL-G: Writing – review & editing, Project administration, Methodology, Formal analysis.
